# Notes of a Case of Acute Yellow Atrophy of the Liver
1Read at a meeting of the Society on January 10th, 1906.


**Published:** 1906-03

**Authors:** F. H. Edgeworth

**Affiliations:** Professor of Medicine, University College, Bristol; Physician to the Bristol Royal Infirmary


### NOTES OF A CASE OF ACUTE YELLOW ATROPHY OF THE LIVER.[1]

BY

F. H. EDGEWORTH, M.B. Cantab., D.Sc. Lond.,

*Professor of Medicine, University College, Bristol; Physician to the Bristol Royal Infirmary.*

---

ACUTE yellow atrophy of the liver is such a rare disease that individual cases are worthy of publication. The following is the only instance which has occurred at the Royal Infirmary in the last eighteen years.

A woman, aged 40, attended the out-patient department on July 8th last, complaining of nausea, anorexia, and occasional vomiting for the preceding week. She had never had any serious illness, and had never been jaundiced before. She had drunk a fair quantity of beer. She was found to be slightly jaundiced, with a normal temperature, a good pulse of 90 per minute, no arterio-sclerotic changes, normal heart and lungs. The vertical extent of hepatic dulness was normal, reaching to the costal margin in the right nipple line. Nothing wrong was detected on palpation of the abdomen—no tumour or distended gall-bladder, or enlarged spleen. A diagnosis of catarrhal jaundice was made. Salicylates and saline purgatives were ordered.

On July 14th, one week later, she again attended. The nausea and anorexia were better, but the jaundice still persisted. The urine contained bile pigments but no albumen.

On July 20th she felt sick again, had pain in the abdomen, and vomited repeatedly; on the following morning she gradually became comatose, and was admitted to the Infirmary about 11 a.m. I saw her at 12 o'clock. She was in a semi-comatose state, with clenched teeth, equal pupils slightly reacting to light, and moving a little when the skin was sharply pinched.

[1] Read at a meeting of the Society on January 10th, 1906.

The skin, of a faint orange colour, was sweating profusely, and the temperature was 99° F. The pulse was regular, of low tension, 134 per minute. The apex of the heart was ½ in. internal to the left nipple line, and no added sounds were present. The lungs were clear down to the bases. The abdomen was flaccid, no enlarged subcutaneous veins were visible. No ascites, or tumour, or other abnormality was discoverable. The spleen was not enlarged. The vertical extent of the hepatic dulness was 2½ in. in the nipple line, the lower edge being a finger's breadth above the costal margin. The urine—a catheter specimen—was bile-stained, acid in reaction, sp. gr. 1023, and contained a fair amount of albumen; it deposited numerous bile-stained granular casts, but no leucin or tyrosin crystals; it contained 2 per cent. of urea.

There was no œdema of the legs; these and the arms were equally and perfectly relaxed. The knee-jerks were equal and exaggerated. There were some doubtful purpuric spots on the arms and legs. The fundi of the eye were pale, with normal vessels, and the optic discs were of a primrose-yellow colour. The patient was given 5 grs. of calomel in a nasal feed. At 3.30 the same afternoon she had a convulsion; twitchings began in the face and spread all over the body. At 5.15 p.m. she had another, and at 7 o'clock a third, in which she died.

On reviewing these phenomena, it would appear that the patient had jaundice for a fortnight, preceded by some gastro-intestinal symptoms; then coma of sudden onset, followed by convulsions, and death within nine hours of its onset. With these symptoms it was found that the liver had slightly diminished in size and that albumen had appeared in the urine.

At the *post-mortem* examination it was found that the brain, heart, lungs, abdomen, and spleen were normal. The uterus was slightly increased in size; it contained no fœtus or evidence of pregnancy. The kidneys were a little enlarged; they peeled well and presented no naked-eye abnormality. The liver was considerably reduced in size and weighed only 32 oz. = ⅔ of normal; it was very flaccid; the capsule, which could be peeled off, was loose and wrinkled; the substance of the liver was of a mottled yellow and red colour, and no normal hepatic tissue could be seen. The bile-ducts were patent and contained no gall-stones. No evidence of phosphorus was detected on distilling portions of the stomach and intestines and their contents in the dark. On microscopical examination it was found that the cardiac muscle-fibres showed some pigmentary degeneration—a change probably quite independent of the fatal disease. There was extensive granular degeneration of the cells lining the convoluted tubes of the kidneys and desquamation of the cells lining Bowman's capsules. This was the cause of the albuminuria and tube-casts in the urine.

In the areas of yellow atrophy in the liver the hepatic cells were granular and their nuclei were hardly visible. The epithelium lining the smaller bile-ducts was proliferating. Round the bile-ducts and vessels in the portal spaces there was some increase of fibrous tissue—due no doubt to the patient's alcoholic intemperance. In the areas of red atrophy the hepatic cells had altogether disappeared. The microscopical examination thus showed evidence of acute necrotic degeneration of the liver cells, with a small amount of cirrhosis. The same poison (? of bacterial origin) which destroyed some and damaged others of the hepatic cells also damaged the secreting cells of the kidneys.

The clinical manifestations of this case were almost, but not quite, typical of the condition. As usual, there was nothing to distinguish the first stage from ordinary catarrhal jaundice. The terminal stage was a little exceptional in that there were no signs of cerebral irritation—headache, restlessness, delirium—preceding the coma; the symptoms were abdominal pain and vomiting. No leucin and tyrosin were found in the urine; but it would seem that these substances are absent in about a third of published cases. The case unfortunately does not shed any light on the causation of this obscure disease. What the poison was, where it was produced, how it affected the brain, whether it accumulated in the body owing to its damaging the renal tissue, are all unknown.

